# Human and bovine tuberculosis knowledge, attitude and practice (KAP) among cattle owners in Ethiopia

**DOI:** 10.1016/j.heliyon.2021.e06325

**Published:** 2021-03-08

**Authors:** Amare Bihon, Solomon Zinabu, Yimer Muktar, Ayalew Assefa

**Affiliations:** a Woldia University, College of Agriculture, Department of Veterinary Medicine, Mersa, Ethiopia; b Samara University, College of Veterinary Medicine, Samara, Ethiopia

**Keywords:** Cattle, Ethiopia, Gondar, KAP, Human TB, Bovine TB

## Abstract

Tuberculosis (TB) is a re-emerging disease occurring worldwide, resulting in multi-billion-dollar loss and human death annually. The situation is worse in developing countries like Ethiopia, where lower knowledge, attitude, and practice (KAP) of the people is poor about the disease. A questionnaire-based cross-sectional study was conducted to assess livestock owners' KAP level towards human and bovine Tuberculosis in Gondar, Ethiopia. A total of 349 study participants were addressed through a face-to-face interview. Descriptive statistics and Pearson's chi-squares analysis were used to analyze the data and observe the association between outcome (KAP level) and predictor variables (sociodemographic characteristics). Out of the 349 respondents, 223 (63.9%) were males, while 126 (36.1%) were females. The KAP measuring interview indicated that 97.4% of the participants are aware of human tuberculosis, while only 84 (24.1%) know about bovine tuberculosis cause and mode of transmission. Inhalation was reported as the main route of transmission for human TB (41.1%) whereas, 50% of the respondent mentioned inhalation, contact, and ingestion of raw animal products as the main route of TB transmission from animal to human. Among those who have heard of bovine tuberculosis, only 56 (66.7%) of respondents consider bovine tuberculosis as a significant threat to public health. The study showed there is a lower KAP on bovine TB among cattle owners in the study area. Therefore, community health education about the impact of the disease, transmission, control, and prevention should be integrated with one health-oriented education and research to eradicate the disease from the country.

## Teaser

Cattle; Ethiopia; Gondar; KAP; Human TB; Bovine TaB

## 1 Introduction

Tuberculosis (TB) is a re-emerging disease occurring worldwide, causing multi-billion-dollar loss and human death annually. The disease affects both humans and animals caused by a group of bacteria called Mycobacterium tuberculosis complex of different species, including Mycobacterium tuberculosis and Mycobacterium bovis (Thoen et al., 2009). M. tuberculosis primarily causes TB in humans, whereas M. bovis predominantly affects cattle (Pal et al., 2014). It is the cause of Zoonotic TB in humans that can spread from infected vertebrate animals to humans (Cosivi et al., 1998; Ashford et al., 2001; Pal, 2007; Pal et al., 2014).

The burden of human TB in Ethiopia is one of the world's highest (Pal et al., 2014; WHO, 2014). The country remains an epicenter for potential zoonotic diseases such as *Bovine tuberculosis* (Grace et al., 2012), putting the public health sector at risk. The exponential growth of the population of the country demanded an increase in animal products. In turn, this scenario resulted in an intensification of dairy and feedlot farms of productive breeds of animals (Ameni et al., 2007; Elias et al., 2008). The situation created a conducive environment for spreading zoonotic diseases like bovine tuberculosis (Ameni et al., 2003). Bovine TB in cattle is manifested throughout different agro-ecological zones of Ethiopia. Its prevalence in cattle ranges from 16.2% to 65.8% in different farming systems (Shitaye et al., 2007). However, a meta-analysis study indicated that the pooled prevalence of bovine tuberculosis in Ethiopia is 5.8%.

In Ethiopia, the prevalence of mycobacterium tuberculosis reaches 0.6%. The mycobacterium complex cause tuberculosis (TB) in various mammalian hosts but exhibit specific host tropisms (Ameni et al., 2011). The Bacterium had demonstrated a potential for reverse zoonoses due to microclimate sharing between humans and animals. The prevalence in this context may not exceed 1% (Ocepek et al., 2005). In Ethiopia, there have been reports demonstrating Mycobacterium tuberculosis infecting bovines (Ameni et al., 2011). A study in central Ethiopia reported that 27% of isolates from grazing cattle were Mycobacterium tuberculosis.

In this particular study area, TB is one of the most prominent health constraints. It is the leading killer of people living with HIV AIDS. Furthermore, bovine tuberculosis in the Gondar town is a substantial public health risk. Its prevalence in different production systems reported in studies (Tintagu Gizaw, 2017) indicated a prevalence of 8.3% from the abattoir-based study. Simultaneously, it was reported to reach 11% in dairy production herds (Mekonnen et al., 2019). Even though the disease poses a substantial public health risk in the Gondar area, people's KAP is not studied.

Studies conducted so far on human and bovine TB in Ethiopia indicated that there is still a gap in KAP about the diseases. A study conducted in Addis Ababa indicated that only 13.9% knew bovine. This indicates that community members living in the capital even have a knowledge gap about the disease. Besides a study conducted in Gondar on high school students measuring their level of understanding about human TB showed that only 59% were knowledgeable about the disease. All these results indicated a knowledge gap to be filled (Hibstu and Bago, 2016; Kidane et al., 2015).

In developing countries like Ethiopia, a low living standard in both animals and humans plays a significant role in bovine tuberculosis transmission between human to human and human to cattle or vice versa (Ameni et al., 2010; Ejeh et al., 2013). Educational efforts were reserved for addressing human to human transmitted TB even though the impact of TB from animal to human is significant. Cattle owners and those in contact with the animal and their products are at risk of acquiring bovine tuberculosis (Ameni et al., 2003). A community based public health education remains the most powerful weapon in promoting awareness among cattle owners. The knowledge about the implication of bovine tuberculosis in humans has to be developed and disseminated adequately. Before planning an educational program, the level of understanding of livestock owners towards the disease have to be measured. With this understanding, a cross-sectional study to assess the community's KAP on human and bovine TB was designed.

## 2 Material and method

### 2.1 Study population and area description

The study involved interviewing household heads who rear cattle and other animals, who have contact with animals and who consume animal products. The study was conducted from February to April 2019 in and around Gondar town. Gondar town is found northwestern part of Ethiopia at 748km away from Addis Ababa and 180km from North East of Bahir Dar. The estimated human population is of the estimated to be 207,044 and the total area of the city covers 5560 ha (CSA, 2007).

### 2.2 Study design and sample size determination

A cross-sectional study was conducted from February to April 2019. Participants were selected using a random sampling approach. From Amhara national, regional state of Gondar town administrative agricultural and rural development office, the cattle owners' list was obtained. The list of all cattle owners was found, a random number was generated to select participant cattle owners. The interview sample size was determined using the methods described by (Yemane, 1967) with finite population correction for proportions formula and using a 5% margin of error at a 95% confidence interval, the total sample size was 349 cattle owners.

### 2.3 Questionnaire design and data collection methods

A close-ended questionnaire was developed which consisted of four parts to measure KAP level of owners were developed based on the common understanding of the disease and literature reviews. The contents of the questions were focused on participants' demographic information, knowledge, attitude, and practice (KAP) measuring questions. The questionnaire was pre-tested and administered through **face-to-face** interviews through house-to-house visits.

The KAP questionnaires main contents focused measuring the participants knowledge about causes, transmission mode, treatment, control and prevention mechanisms of human and bovine TB. Besides, we introduced questions that assess the attitudes and practices of participants about habit of raw meat/milk consumption, husbandry/management practices, herd size and structure owned, watering/feeding, production system, presence of contact between human and cattle, and known current or previous history of TB status in their households was recorded.

### 2.4 Ethics statement

According to the National Research Ethics Review Guideline of Ethiopia, this research doesn't require formal ethical approval. However, we had verbal consent with participants for the right of confidentiality of information they provide.

### 2.5 Data management and analysis

The collected data were cleaned, checked entered using Epi data software. Then, it was exported to Microsoft Excel and analyzed by SPSS version 20 software package. KAP for each participant was calculated by giving a score. If a participant correctly answered questions, we gave one, if not we gave zero for any wrong answer. All the correct answerers were added and divided by the number of participants; and multiplied by 100 to determine the mean percentage level of knowledge, attitude and practices questions. For each participant who scored above the average we classify them to have good KAP, while those who scored below the average, we group them to have poor KAP. For association analysis between KAP level and socio demographic predictor variables, respondents were categorized as those who had good KAP or those who had poor KAP level based on score greater than or equal to the mean value assigned as having good KAP, and the score less than the mean value as poor KAP level. The relationships between the predictor variable (age, gender, marital status, educational status, occupation, residence) with KAP scores were examined using Pearson's chi-square value.

## 3 Results

### 3.1 Socio-demographic characteristics of the participants

A total of 349 cattle owners participated in this study. Among them 223 (63.9%) were males, while 126 (36.1%) were females. Most of the respondents (39.5%) were between 18 to 30 years old. Regarding the educational status, the highest number (25.2%) of the respondents were illiterate. Majority of the respondents (40.4%) were engaged cattle rearing practices. Regarding residential area, 236 (67.6%) study participants live in rural peasant associations while the rest were in city administration (Table 1).

**Table 1:** Socio-demographic characteristics of respondents.

Variable	Category	Frequency (%)
Age	18–30	138 (39.5)
	31–40	95 (27.2)
	41–50	63 (18.1)
	>51	53 (15.2)
Gender	Male	223 (63.9)
	Female	126 (36.1)
Marital status	Married	258 (73.9)
	Single	91 (26.1)
Educational status	Illiterate	88 (25.2)
	Write and read-only	53 (15.2)
	Less than grade 8	77 (22.1)
	Grade 8-12	76 (21.8)
	Above grade 12	55 (15.8)
Current occupation	Farmer	141 (40.4)
	Student	63 (18.1)
	Employee	24 (6.9)
	Merchant	87 (24.9)
	Labor	34 (9.7)
Residence	Rural	236 (67.6)
	Urban	113 (32. 4)
**Total**		**349 (100.0)**

### 3.2 Species of animals owned, purpose and husbandry system

Majority of the participants 232 (66.5%) owned local cattle breeds (*Boss indicus*), and the remaining 117 (33.5%) had cross breed between Boss indicus and *Boss taurus*) cattle. Besides, 194 (55.6%) keep cattle only, while the rest rear cattle and other livestock. The primary purpose/reasons to keep cattle were milk production (42.75%), milk and draft (36.5%), and meat production. Regarding husbandry practices of cattle, 168 (48.1%) graze their animals free in the fields, 110 (31.5%) keep their animal in intensive management, and the rest 71 (20.3%) respondents practice semi-intensive management systems (Table 2).

**Table 2:** Species of animal owned, the purpose of rearing and management system.

Variables	Category	Frequency (%)
Species of animal owned	Cattle only	194 (55.6)
	Cattle and sheep	101 (28.9)
	Cattle, sheep, and goat	41 (11.7)
	Cattle, sheep, goat, and poultry	13 (3.7)
Breeds of cattle	Local	232 (66.5)
	Cross	117 (33.5)
Purpose of rearing	Milk	149 (42.7)
	Meat	28 (8.0)
	Milk and draft	127 (36.4)
	Milk, meat, and draft	45 (12.9)
Herd size	<10	251 (71.9)
	10–20	63 (18.1)
	>20	35 (10.0)
Management system	Free grazing	168 (48.1)
	Intensive (indoor keeping)	110 (31.5)
	Semi-intensive	71 (20.3)
**Total**		**349(100.0)**

### 3.3 Knowledge of respondents towards human and bovine tuberculosis

From the total participants, 97.4% of them have knowledge on human TB while the rest 2.6% have no information about the disease. In contrast most of the participants (75.9%) had never heard about bovine TB. Misperceptions such as bad weather (both hot and cold) and genetically from parents were thought to be associated with the disease. Most of the respondents mentioned that TB patients (40.0%) and radio/TV (41.1%) as the primary source of information on human and bovine TB. Concerning the modes of human TB transmission, 130 (41.1%) said that M. tuberculosis can be transmitted by inhaling exhaled air when a person with TB coughs, sneezes and speaks/droplet transmission. Among the respondents who had information about bovine TB, 42.9% thought that the ingestion of raw animal products (milk and meat) as the mode of transmission from animals to humans (Table 3).

**Table 3:** Participants understanding and information source about cause and transmission of human and bovine tuberculosis.

Variable	Category	Human TB Frequency (%)	Bovine TB Frequency (%)
Heard of the diseases	Yes	340 (97.4)	84 (24.1)
	No	9 (2.6)	265 (75.9)
Source of information	Newspaper	17 (5.0)	4 (4.8)
	Radio/TV	71 (20.9)	35 (41.7)
	TB patient	136 (40.0)	12 (14.3)
	Health institute	37 (10.9)	------
	School	6 (1.8)	------
	Family	19 (5.6)	------
	Multiple sources	54 (15.9)	33 (39.3)
Cause of the disease	Bad weather	107 (31.7)	7 (8.3)
	Microorganism (bacteria)	154 (45.3)	53 (63.1)
	Genetically from parents	25 (7.4)	16 (19.0)
	Did not know	54 (15.9)	8 (9.5)
Way of transmission	Inhalation	130 (41.1)	4 (7.1)
	Contact	21 (6.6)	-------
	Ingestion of raw milk and meat	-------	24 (42.9)
	Inhalation, contact and ingestion (raw milk and meat)	31 (9.8)	28 (50.0)
	Inhalation and contact	112 (35.4)	------
	Did not know	22 (7.0)	------
**Total**		**349 (100.0)**	**349 (100.0)**

The majority of respondents believed that human TB is a curable disease. 298 (94.3%) of the participants stated that drugs from health center are the best treatments for TB. The highest number of respondents stated that TB transmissions could be preventable. Furthermore, 99 (31.7%) of the participants mentioned that covering mouth and nose when coughing and sneezing, avoid sharing of utensils, and separation of the patient room as a commonly used method for preventing the spread and transmission of TB. Moreover, 51 (16.7%) respondents mentioned that the spread of TB could be reduced through vaccination to human and cattle even though vaccination in for bovine TB is not a common practice in the area. Among participants who had awareness about TB in humans and animals, 30.9% witnessed TB among their family members or friends. Regarding the type of TB observed, 78.2% referred to a pulmonary form and 21.9% to an extrapulmonary form of TB. About the TB patients' treatment history, 92% of them took modern drugs given by the health center (Table 4).

**Table 4:** Knowledge of respondents related to control and prevention of human tuberculosis.

Indicative variable	Category	Frequency (%)
Do you know TB a curable disease?	Yes	316 (92.9)
	No	24 (7.1)
In what ways TB can be cured?	Herbal remedy	12 (3.8)
	A specific drug is given health center	298 (94.3)
	Home rest and pray	6 (1.9)
Do you know TB preventable?	Yes	312 (91.8)
	No	28 (8.2)
What methods can be used to reduce transmission of TB	††	99 (31.7)
	Covering mouth and nose	58 (18.6)
	Vaccination	51 (16.7)
	Isolating patients	44 (14.1)
	Avoid sharing of utensils	41 (13.1)
	Early treatment of patients	8 (2.6)
	Eating only cooked animal products	7 (2.2)
	Eating a balanced diet	4 (1.3)
Is there a TB patient family member or a friend?	Yes	105 (30.9)
	No	235 (69.1)
What type of TB was observed in family/friend	Pulmonary	82 (78.1)
	Extrapulmonary	23 (21.9)
Were they treated?	Yes	100 (95.2)
	No	5 (4.8)
What type of treatment given?	Traditional	8 (8.0)
	Modern	341 (92.0)

††Covering mouth in public place, avoid sharing of utensil and separating a patient room.

### 3.4 The attitude of respondents towards human and bovine tuberculosis

Regarding the attitude towards people with TB disease, 233 (68.5%) of the respondents feel compassion and desire to help, while 57 (16.8%) feel compassion, but they tend to stay away, 31 (9.1%) respondents said they have fear of contracting the disease and will not get closer to patients, and the remaining 19 (5.6%) participants have no particular feelings towards TB patients. Most of the respondents, 143 (42.6%), said that most of the community usually segregate TB patients. Among the participants, 48 (14.1%) responded that TB affects only poor people. The highest proportion of the respondents, 212 (62.4%), did not consider the consumption of the raw animal products (milk and meat) to pose a risk for exposure to bovine tuberculosis, whereas 31 (9.1%) respondents were not sure about it. Two-thirds of respondents (67.6%) stated that vaccination against TB would protect anyone from TB disease (Table 5).

**Table 5:** Attitude of respondents towards tuberculosis in the study area.

Indicative variables	Category	Frequency (%)
What do you feel towards TB patients?	Feel compassion and desire to help	233 (68.5)
	Feel compassion and stay away from them	57 (16.8)
	Fear them because they may transmit TB	31 (9.1)
	Have no particular feeling	19 (5.6)
How you regarded TB patient	Most people reject them	143 (42.6)
	Most people are friendly, but gradually reject them	32 (9.5)
	Mostly support them	93 (27.7)
	Other††	68 (20.2)
Do you think that TB affects only poor people?	Yes	48 (14.1)
	No	292 (85.9)
Do you think the consumption of raw animal product exposes to TB?	Yes	97 (28.5)
	No	212 (62.4)
	Not sure	31 (9.1)
Do you think that TB has a negative impact?	Yes	285 (83.8)
	No	51 (15.0)
	Did not know	4 (1.2)
Do you think that vaccination against TB is avail?	Yes	148 (43.5)
	No	192 (56.5)
Do you think vaccination will protect anyone against TB?	Yes	100 (67.6)
	No	18 (12.2)
	Have no idea	30 (20.3)

†† did not know their feeling, not sure whether they help or not, did not give special attention.

### 3.5 The practice of participants towards bovine tuberculosis

From the total participants, 10.3% of the households practice raw milk consumption, while 41.3% boil fresh milk before consumption. Most respondents boil milk by fearing milk-borne diseases, while 42.2% of the household heads boil milk for cultural reasons. More than one-fourth (27.5%) of participants responded that they share the same watering point with cattle. 28 (8%) respondents stated they share the same house with their animals. Nearly three-fourths (74.1%) of the respondents advised TB patients to checkup in health centers. Nine among ten (90.3%) respondents will go to the hospital if they think they had been infected with TB, and 23 (6.8%) go to the pharmacy, whereas the rest would prefer visiting traditional healers (Table 6).

**Table 6:** General practice of study participants towards TB in the study area.

Indicative variable	Category	Frequency (%)
The habit of milk drinking	Boiled/pasteurized	144 (41.3)
	Raw milk (Including fresh milk and yogurt)	31 (10.3)
	Both	169 (48.8)
Reason for milk boiling	Fear of milk born disease	181 (57.8)
	Culture	132 (42.2)
Habit of meat consumption	Raw meat	15 (4.3)
	Cooked meat	249 (71.3)
	Both	85 (24.4)
Sharing of the same water source with animal	Yes	96 (27.5)
	No	253 (72.5)
Sharing of a house with animal	Yes	28 (8.0)
	No	321 (92.0)
What do you do if you see TB patients?	Advise them to go to the hospital	252 (74.1)
	ignore them	88 (25.9)
What do you do if you had infected with TB?	Go to hospital	307 (90.3)
	Go to pharmacy	23 (6.8)
	Go to a traditional healer	10 (2.9)
**Total**		**349 (100.0)**

### 3.6 Knowledge, attitude, and practice towards the zoonotic potential of bovine tuberculosis

Among the study participants who had information about bovine tuberculosis 84 (24.1%), 56 (66.7%) regarded bovine tuberculosis as a significant public health threat. More than half (60.7%) of participants stated that raw milk and meat as the source of bovine tuberculosis. However, more than thirty percent (33.3%) of respondents think bovine TB affects animals only. Most of the respondents (69.6%) mentioned that using cooked meat and boiled milk reduces the transmission of bovine tuberculosis from animals to humans (Table 7).

**Table 7:** Knowledge, attitude and practice of study participant towards the zoonotic potential of BOVINE TUBERCULOSIS in the study area.

Indicative variables	Category	Frequency (%)
Is TB of cattle communicable to a human?	Yes No	56 (66.7) 28 (33.)
Do you consider raw milk and meat as a source of BOVINE TUBERCULOSIS?	Yes No	51 (60.7) 33 (39.3)
Method to reduce BOVINE TUBERCULOSIS transmission from animal to human	Separating house of human and animal	5 (8.9)
	Using cooked animal product	39 (69.6)
	Keeping the health of an animal	4 (7.1)
	All of them	8 (14.3)

### 3.7 Factors associated with KAP level of the respondent towards human and bovine tuberculosis

KAP level was calculated by scoring one for a correctly provided answer and zero for the wrong answer. The respondents' average score was categorized as good KAP and poor KAP based on a KAP score of ≥11.02 ± 3.575 as good and a KAP score ≥ of 3.07 ± 2.058 as poor KAP for human TB. Similarly, for bovine TB, the KAP score ≥11.02 ± 3.575 regarded as good KAP, while the score <3.07 ± 2.058 was categorized as poor KAP. Based on this calculation, about 178 (51%) and 65 (18.6%) respondents had good KAP levels for human TB (Table 8) and Bovine TB (Table 9), respectively. There was a significant association between KAP scores and the respondent's age (p < 0.05). The highest proportion of respondents 25 (7.1%) having good KAP level towards bovine tuberculosis was in the age group of 31–40, while respondents with good KAP towards human TB were in 18–30 age category. Educational status and current occupation were associated with KAP scores (p < 0.05). The study participants' residence was associated with KAP scores on bovine TB (c2 = 10.361, p < 0.05).

**Table 8:** Factor associated with cattle owners KAP towards human TB.

Variables	KAP level		x^2^	P-value
	Good KAP	Poor KAP		
**Age**				
18–30	58 (16.6%)	80 (22.9%)		
31–40	52 (14.9%)	43 (12.3%)	8.266	0.041
41–50	39 (11.1%)	24 (6.8%)		
>51	29 (8.3%)	24 (6.8%)		

**Gender**				
Male	110 (31.5%)	113 (32.4%)	0.694	0.405
Female	68 (19.5%)	58 (16.6 %)		
**Marital status**				
Married	134 (38.4%)	124 (35.5%)	0.346	0.556
Single	44 (12.6%)	47 (13.5%)		
**Educational status**				
>Grade 12	43 (12.3%)	12 (3.4%)		
Grade 8-12	41 (11.7%)	35 (10.0%)		
< Grade 8	40 (11.5%)	37 (10.6%)		
Write and read only	34 (9.7%)	19 (5.4%)	48.369	0.000∗∗∗
Illiterate	20 (5.7%)	68 (19.5%)		

**Current occupation**				
Farmer	60 (17.2%)	81 (23.2%)		
Merchant	55 (15.8%)	32 (9.2%)		
Student	35 (10.0%)	28 (8.0%)		
Employee	19 (5.4%)	5 (1.4%)	25.552	0.000∗∗∗
Labor	9 (2.6%)	25 (7.2%)		

**Residence**				
Rural	117 (33.5%)	119 (34.1%)	0.594	0.441
Urban	61 (17.5%)	52 (14.9%)		
**Total**	**178(51%)**	**171(49%)**		

Key: Good KAP: KAP score ≥11.02 ± 3.575, and KAP score ≥3.07 ± 2.058 respectively for human and bovine TB KAP. Poor KAP: KAP score <11.02 ± 3.575 and KAP score <3.07 ± 2.058 respectively for human and bovine TB KAP.

**Table 9:** Factor associated with cattle owners KAP towards bovine TB.

Variables	KAP level		x^2^	P-value
	Good KAP	Poor KAP		
**Age**				
18–30	19 (5.4%)	119 (34.1%)		
31–40	25 (7.1%)	70 (20.1%)	8.839	0.032∗
41–50	15 (4.3%)	48 (13.8%)		
>51	6 (1.7%)	47 (13.5%)		

**Gender**				
Male	38 (10.9%)	185 (53.0%)	1.023	0.312
Female	27 (7.7%)	99 (28.4%)		

**Marital status**				
Married	53 (15.2%)	205 (58.7%)	2.402	1.21
Single	12 (3.4%)	79 (22.6%)		

**Educational status**				
>Grade 12	20 (5.7%)	35 (10.0%)		
Grade 8-12	17 (4.9%)	59 (16.9%)		
< Grade 8	15 (4.3%)	62 (17.8%)		
Write and read only	10 (2.9%)	43 (12.3%)		
Illiterate	3 (0.9%)	85 (24.4%)	25.604	0.000∗∗∗

**Current occupation**				
Merchant	25 (7.1%)	62 (17.8%)		
Farmer	15 (4.3%)	126 (36.1%)		
Student	12 (3.4%)	51 (14.6%)		
Employee	11 (3.2%)	13 (3.7%)	27.175	0.000∗∗∗
Labor	2 (0.6%)	32 (9.2%)		

**Residence**				
Rural	33 (9.5%)	203 (58.2%)	10.361	0.001∗∗
Urban	32 (9.1%)	81 (23.2%)		
**Total**	**65(18.6%)**	**284(81.4)**		

## 4 Discussion

The present study revealed that almost all cattle owners (97.4%) have information about human TB, while less awareness about bovine tuberculosis (24.1%). This result with regard to M. tuberculosis in humans was in agreement with a study conducted in Addis Ababa and in southern Ethiopia that reported 99.5% KAP level (Kidane et al., 2015) 99.6% (Hibstu and Bago, 2016) respectively, who found a profound awareness about human TB among high school students. Nevertheless, Romha et al. (2014) indicated a lower (29.7%) awareness of bovine tuberculosis among cattle owners in the southern part of Ethiopia. Likewise, Getahun and Eshetu (2018) reported that 69.0% of respondents have no information about bovine tuberculosis among the community in the Gambella region of Ethiopia. The current study revealed a higher proportion of bovine tuberculosis knowledgeable respondents than the report of Kidane et al. (2015), explaining 13.9% of knowledgeable high school students in Addis Ababa, Ethiopia. On the contrary, different studies showed a higher proportion of knowledgeable respondents about bovine tuberculosis (Kuma et al. (2013) in Jimma zone in South West Ethiopia, Ameni and Erkihun (2007) in Adama, Central Ethiopia, Munyeme et al. (2010) in Zambia and Addo et al. (2011) in China reported 45.6%, 35%, 39.6%, and 88% respectively). More than 20% of cattle owners said that they get information and awareness about the disease from radio/television (TV) both national and local channels. Similarly, Hoa et al. (2009) reported (64.6%) respondents get information from television. This may be due to the recent attention given by the government and NGOs operating in Ethiopia. These organizations always air information on these diseases on Tv and radio to create awareness. On the other hand, Yadav et al. (2006) described that neighbors, friends, and family members as a significant source of information in India. Thus, different intervention means and efforts are suggested to consider each setting's peculiar nature and target group (Hoa et al., 2009). In this study, the greater awareness about human tuberculosis could reflects remarkable educational efforts towards human TB through multiple information sources, participation large number of multicultural respondents in animal production, health, and husbandry. Despite a higher proportion of the study participants having information about human TB, more than half (56.7%) had little knowledge about the disease's cause. More than half (63.1%) of the respondents mentioned germ/bacteria is the actual cause of bovine tuberculosis. However, misperceptions such as bad weather (both cold and hot air) and genetically from parents were implicated as a cause of human and bovine TB. This finding is in line with Gebremariam et al. (2011), Bati et al. (2013) and Getahun and Eshetu (2018), who reported similar misperceptions among the general community in Addis Ababa and Gambella region, southwestern part of Ethiopia.

We found that the zoonotic potential of bovine tuberculosis was not well known by cattle owners. Among those who have an awareness of bovine tuberculosis, (33.3%) believed that no transmission of TB from animal to human occurs. In line with this, Kidane et al. (2015) reported similar results among high school students in Addis Ababa. Likewise, Bati et al. (2013) and Romha et al. (2014) highlighted that only 22.9% and 16.6% of respondents believed the fact that TB can be acquired from animals, respectively. Apart from the variation due to the study population's difference with multicultural practice in the respective study areas, it also implicates the wide knowledge gap among the general community regardless of age group.

From respondents who had information about bovine tuberculosis, 42.9% stated that the ingestion of raw animal products (milk and meat) as the mode of bovine tuberculosis transmission of zoonotic TB. Similarly, different studies reported the culture of raw milk consumption in Ethiopia as a potential transmission way of M. bovis to humans (Ameni and Erkihun, 2007; Bati et al., 2013; Romha et al., 2014). More than half (57.8%) of study participants boil milk due to fear of milk borne disease such as tuberculosis, brucellosis and E. coli. Similar but much higher findings were reported by Kidane et al. (2015) in Addis Ababa and Getahun and Eshetu (2018) in the Gambella region, southwest Ethiopia with 66.2% and 90.9% of respondents, boil milk due to fear of milk-borne diseases, respectively. This proves awareness of people who practice boiling improves disease prevention practice. Less than half (41.1%) of the respondents recognized that human TB could be transmitted through inhalation of exhaled air when a person with TB coughs, sneezes, speaks or sings. This result was inconsistent with the studies conducted in Ethiopia's different areas (Legesse et al., 2010; Abebe, 2010) and Selangor (Noremillia and Haliza, 2015), who reported 80.8% and 96% respectively. The inconsistency could be due to the variability of information and study population.

Significant portions (30.9%) of respondents have closely witnessed the presence of TB cases in relatives or friend typical clinical signs include coughing, fever and loss of appetite. More than three-fourth (78.2%) of participants referred to a pulmonary form and 21.9% to an extrapulmonary form regarding the type of TB observed. However, the rates were higher than those reported by Kidane et al. (2015) in Addis Ababa and Getahun and Eshetu (2018) in southwest Ethiopia, where 21.7% and 19.3% reported pulmonary forms, respectively. Regarding the TB patients' treatment history, 92.0 % of them took a modern drug given by health centers. This is in line with a study conducted in southwestern Ethiopia (Getahun and Eshetu, 2018).

Most of the participants responded that TB is curable with modern drugs, covering their mouth and nose when coughing and sneezing, avoids sharing of utensils and separating patient room as important prevention and control approach indicated that awareness of the study community about the appropriate treatment and prevention measure of the disease could play a significant role in reducing the spread of the disease (Bati et al., 2013).

More than two-thirds (68.5%) of the respondents feel compassion and desire to help TB patients. This finding was higher than the study reported by Hibstu and Bago (2016) in southern Ethiopia. More than ten percent (14.1%) of the entire study participants stated that TB affects only poor people. This was in line with the finding in rural Ethiopia by Yimer et al. (2009). Nine among ten (90.3%) respondents would go to a health facility if they think TB had infected them, and the rest would prefer to find other self-treatment options like herbs and to visit traditional healers. This result was nearly similar a study conducted in southern Ethiopia (Hibstu and Bago, 2016).

The study participant's educational status for awareness of TB in humans and animals was significantly associated with the KAP score (P < 0.05). All respondents with grade eight and above educational level had good KAP of TB in humans and animals. The possible reason could be as education increases, people would have acquired better information access about the diseases. This result is consistent with previous reports in Ethiopia (Mesfin et al., 2005; Bati et al., 2013). The finding of this study revealed that farmers and merchants were more knowledgeable than the rest of the study groups.

## 5 Conclusions

Even though a relatively good understanding of TB was observed compared to previous studies, the KAP level was not adequate. 97.4% of the participants know human tuberculosis about its cause, transmission, symptoms and prevention approaches, while 24% know bovine tuberculosis cause and transmission mode. Respondents had a lower level of understanding of the zoonotic potential of bovine TB. It is an indication that the public health wing of the Veterinary service provider of the country should develop education programs with regard to zoonotic diseases such as tuberculosis, brucellosis, anthrax and food safety. If the country needs to eradicate such disease with a substantial public health impact, the plan should start from grass root level by creating awareness to livestock owners and animal product consumers. Community health education about the impact of the disease, transmission, control, and prevention should be integrated with one health-oriented education and research to eradicate tuberculosis and other zoonotic diseases from the country.

## Declarations

### Author contribution statement

Amare Bihon: Conceived and designed the experiments; Performed the experiments; Analyzed and interpreted the data; Wrote the paper.

Solomon Zinabu: Performed the experiments; Analyzed and interpreted the data; Wrote the paper.

Yimer Muktar and Ayalew Assefa: Analyzed and interpreted the data; Wrote the paper.

### Funding statement

This research did not receive any specific grant from funding agencies in the public, commercial, or not-for-profit sectors.

### Data availability statement

Data included in article/supplementary material/referenced in article.

### Declaration of interest statement

The authors declare no conflict of interest.

### Additional information

No additional information is available for this paper.
